# Comprehensive analysis of bioactive compounds in *Crocus sativus* stamens extracts by HPLC-DAD: investigating antidiabetic activity via *in vitro*, *in vivo*, and molecular docking simulation

**DOI:** 10.3389/fchem.2024.1419120

**Published:** 2024-07-12

**Authors:** Samira Mamri, Nour Elhouda Daoudi, Abedellah Baraich, Sabir Ouahhoud, Amine Khoulati, Mohammed Choukri, Abdeslam Asehraou, Mohamed Bnouham, Bassem Jaouadi, Abdelkrim Abousalham, Fakhreldeen Dabiellil, Ahmad Mohammad Salamatullah, Ennouamane Saalaoui

**Affiliations:** ^1^ Laboratory of Bioresources, Biotechnology, Ethnopharmacology and Health, Department of Biology, Faculty of Sciences, Mohammed the First University, Oujda, Morocco; ^2^ Higher Institute of Nursing Professions and Health Techniques, Oujda, Morocco; ^3^ Laboratory of Biological Engineering, Faculty of Sciences and Technology, University Sultan Moulay Slimane, Beni Mellal, Morocco; ^4^ Faculty of Medicine and Pharmacy, University Sultan Moulay Slimane Beni Mellal, Beni Mellal, Morocco; ^5^ Faculty of Medicine and Pharmacy of Oujda, Mohammed First University, Oujda, Morocco; ^6^ Biochemistry Laboratory, Central Laboratory Service—University Hospital Center, Oujda, Morocco; ^7^ Microbial Biotechnology and Enzyme Engineering Laboratory (LBMIE), Center for Biotechnology of Sfax (CBS), University of Sfax, Sfax, Tunisia; ^8^ Institute of Chemistry and Molecular and Supramolecular Biochemistry (ICBMS), Enzymatic Engineering, Biomimetic Membranes, and Supramolecular Assemblies (GEMBAS), Courrier d'Entreprise à Distribution Exceptionnelle, University Lyon, Villeurbanne, France; ^9^ Department of translation, Faculty of Arts, University of Khartoum, Khartoum, Sudan; ^10^ Department of Food Science and Nutrition, College of Food and Agricultural Sciences, King Saud University, Riyadh, Saudi Arabia

**Keywords:** α-amylase, stamens, Crocus sativus, peak blood glucose, postprandial hyperglycemia

## Abstract

**Introduction:** Medicinal herbs used in traditional diabetes treatment are a rich source of anti-diabetic compounds. Pancreatic α-amylase inhibitors offer an effective strategy to reduce postprandial hyperglycemic levels via control of starch degradation. In this context, our study for the first time investigates the effect of *Crocus sativus* stamens extracts on α-amylase inhibition.

**Material and methods:** The hydromethanolic and hydroethanolic extracts were obtained by macerating the dried stamen powder with methanol/water or ethanol/water, respectively. The total phenolic content of the stamen extracts was assessed using the Folin-Ciocalteu reagent method, while the total flavonoid content was determined using the Aluminum Chloride method. Phytochemicals were further quantified and identified using HPLC-DAD. For evaluation of hypoglycemic activity, *in vitro* α-amylase enzyme inhibition was calculated. The results were confirmed *in vivo* using an oral starch tolerance test in both normal and diabetic rats.

**Results:** Our findings demonstrated a higher level of polyphenols and flavonoids in the hydroethanolic extract. Important flavonoids found were kaempferol, rutin, and vanillic acid, while prominent carotenoids contained trans- and cis-crocins. The *in vitro* study showed that both hydromethanolic and hydroethanolic extracts had considerable inhibitory effects, with maximum inhibitions of approximately 83% and 89%, respectively. *In vivo* tests indicated that both extracts effectively lowered peak blood glucose and area under the curve in both normal and diabetic rats following oral starch treatment. The obtained results are also supported by a docking study.

**Conclusion:** These findings imply that *C. sativus* stamens possess a distinctive capability to reduce postprandial blood glucose levels. This effect is likely mediated through the inhibition of α-amylase, presenting a novel dietary avenue for managing diabetes.

## 1 Introduction

The utilization of medicinal plants has gained widespread popularity and is increasingly recognized for its significance in both health and the economy. Herbal treatments are employed for various ailments, with diabetes, being the most prevalent endocrine disease, among the conditions addressed by these natural remedies (N’doua et al., 2016). Diabetes is a metabolic disorder distinguished by persistent hyperglycemia resulting from impaired insulin secretion, insulin action (sensitivity), or a combination of the two (Ozougwu et al., 2013). Diabetes is now responsible for 5.2 million fatalities worldwide, with a mortality rate of 82.4 per 100,000 people. Moreover, diabetes is recognized as a significant risk factor for the onset of cardiovascular disease (CVD), the primary cause of mortality among individuals with diabetes (Benjamin et al., 2018). Numerous studies have demonstrated that elevated postprandial hyperglycemia heightens the risk of microvascular and macrovascular complications in individuals with diabetes (Fan, Glovaci, and Wong, 2019). Therefore, the management of diabetes, particularly by minimizing postprandial glycemia, constitutes a crucial aspect of the treatment of type II diabetes aimed at preventing vascular complications (Ceriello et al., 2004). This objective can be attained by inhibiting carbohydrate-hydrolyzing enzymes, specifically α-amylase and α-glucosidase, which play crucial roles in carbohydrate digestion. α-Amylase is responsible for breaking down long-chain carbohydrates, while α-glucosidase is involved in the conversion of starch and disaccharides into glucose (Nair, Kavrekar, and Mishra, 2013). The current therapeutic approach for managing diabetes involves strict dietary measures and the use of oral antidiabetic drugs. However, this solution proves to be costly, particularly considering the limited purchasing power of populations in many developing countries, such as Morocco. The elevated expenses associated with conventional treatments compel diabetic patients to turn to traditional remedies (Lotfi et al., 2017). The kingdom of plants offers a vast area for the discovery of natural oral hypoglycemic medicines that are effective and cause minimal to no adverse effects (Tundis, Loizzo, and Menichini, 2010). Thus, naturally occurring alpha glucosidase and alpha amylase inhibitors derived from plants present a desirable method for managing hyperglycemia (Tundis, Loizzo, and Menichini, 2010).

In Morocco, *Crocus sativus* L. (Iridaceae) is known to have medicinal proprieties (Khan et al., 2020). It has been cultivated and harvested for centuries for cosmetics, as a culinary coloring and flavoring agent, and as a natural treatment (Schmidt, Betti, and Hensel, 2007). Furthermore, a pharmacological investigation revealed that *C. sativus* stigma had significant effects on diabetes (Sabir et al., 2018). Nevertheless, 1 kg of flowers generates only 15 g of spices. This means that more than 90% of the plant material harvested is discarded (Smolskaite et al., 2011). Recent research has revealed that saffron tepal extract has potential therapeutic benefits like renotoprotective (MAMRI et al., 2022)**,** antioxidant (Ouahhoud et al., 2022), antifungal (Khoulati et al., 2019) and antidiabetic (Sabir et al., 2018) activities. Nevertheless, the pharmacological investigation of *C. sativus* stamens has received minimal attention. *Crocus sativus* stamens have been the subject of some recent scientific investigations aimed at assessing or verifying their advantageous biological effects on a range of diseases. It has been shown that stamens possess a remarkable capability for antioxidants (Montoro et al., 2012) and protective effect against nephrotoxicity (MAMRI et al., 2022). Hence, the aim of the present study is to assess the control of postprandial glycemia through the administration of *C. sativus* stamens extracts. The goal is on elucidating potential mechanisms during the absorptive process, with a specific focus on the activity of alpha-amylase, a pivotal enzyme in carbohydrate digestion.

## 2 Materials and methods

### 2.1 Chemicals and reagents

All the chemicals used in this study are of analytical quality. Sucrose and starch powders were procured from Sigma-Aldrich in Germany. Acarbose and dinitrosalicylic acid were obtained from Sigma-Aldrich in China. The α-amylase enzyme was purchased from Sigma-Aldrich in the United States. Alloxan monohydrate (98% purity) was purchased from ACROS Organics. The glucose oxidase-peroxidase (GOD-POD) kit was acquired from Biosystems in Barcelona, Spain.

### 2.2 Plant material collection

Plants of *C. sativus* were produced on a farm in Taliouine (30° 31′54″north, 7° 55′25″west, Southern Morocco). *Crocus sativus* from this location was grown without the use of any chemicals. Professor Botanist Fennane Mohammed of Morocco’s Scientific Institute of Rabat identified the plant botanically. Three specimens of the plant were registered in the plant section of the University Herbarium Mohammed First, Oujda, Morocco (HUMPOM), with the voucher number (HUMPOM210).

Stamens were manually harvested during late October and early November. Subsequently, the stamens underwent oven-drying at 37°C for a duration of 4 h. The dried stamens were then ground using an automatic grinder and stored as stamen powder at −20°C until required for further use.

### 2.3 Extracts preparation

#### 2.3.1 Preparation of hydroethanolic extract

For the hydroethanolic extract (EthOH) preparation, the stamen powder underwent maceration in a mixture of ethanol and water (80/20, v/v). The plant powder was added at a ratio of 2 g per 50 mL of solvent, and the maceration process lasted 24 h with continuous stirring. This procedure was conducted in darkness and at room temperature (25°C). Subsequently, the solvent was filtered using a 0.45 µm filter. This extraction process was repeated three times, and the combined hydroethanolic phase was then subjected to drying using a rotary evaporator at 40°C. Finally, the resulting dry hydroethanolic extract was stored at −20°C. The various concentrations were obtained from this extract dissolved in distillate water.

#### 2.3.2 Preparation of hydromethanolic extract

For the hydromethanolic extract (MethOH) preparation, 2 g of stamen powder was macerated in 50 mL of methanol and water (80/20, v/v) for 24 h with stirring, in darkness and at room temperature (25°C). After the extraction, the solvent was filtered through a 0.45 µm filter. This process was repeated three times, and the total hydromethanolic phase obtained was dried using a rotary evaporator at 40°C. Finally, the resulting dry hydromethanolic extract was stored at −20°C. The various concentrations were obtained from this extract dissolved in distillate water.

$$\text{Extraction }\text{Yield }\left(\%\right)=\frac{\text{Weight}\;\text{of}\;\text{extract}\;\text{recovered}\;\left(g\right)}{\;\text{Weight}\;\text{of}\;\text{dry}\;\text{matter}\;\left(g\right)}\times 100$$

### 2.4 Determination of total phenolic

The total phenolic content in various stamen extracts was determined using the Folin-Ciocalteu method, following the procedure outlined by Lamien-Meda et al. (Lamien-Meda et al., 2008) with some adjustments. To quantify total polyphenols, a calibration curve was generated from different dilutions of gallic acid (concentration: 1 mg/L) in distilled water.

Stamen extracts were prepared for the analysis, with each extract sample having a concentration of 10 mg/mL. In individual test tubes, 500 μL of each extract sample was mixed with 1.75 mL of distilled water, and then 250 μL of 10% Folin Ciocalteu reagent was added. This mixture was incubated at room temperature in the dark for 5 min. Following this, 500 μL of a 20% Na2CO3 solution was introduced to each test tube. The tubes were then incubated at room temperature for 30 min, and the absorbance of the resulting mixture was measured at 725 nm using a UV-visible spectrophotometer (UV-Visible spectrophotometer T80+).

The regression equation derived from the gallic acid calibration curve. The concentration was expressed in micrograms of equivalent gallic acid per Gram of extract (µg EAG/g extract).

### 2.5 Determination of total flavonoid

The method described by Zhishen et al. (1999) was used to assess flavonoid content using aluminum trichloride reagent (AlCl_3_) and sodium hydroxide (NaOH). Flavonoids react with aluminum trichloride to produce a yellow complex, while sodium hydroxide forms a pink complex with absorption in the 510 nm wavelength range.

In the experimental procedure, the following components were successively introduced into a test tube: 500 μL of the extract, 1.5 mL of distilled water, and 150 μL of a 5% NaNO_2_ solution. After a 5-min interval, 150 μL of AlCl_3_ (10%) was added to the mixture. Furthermore, 6 minutes later, 500 μL of NaOH (4%) was introduced. The absorbance of the resulting mixture was directly measured at a wavelength of 510 nm using a UV-visible spectrophotometer against the blank.

The total flavonoid concentrations in each extract sample were calculated based on the regression equation obtained from the calibration curve, which was constructed using a rutin standard (concentration: 1 mg/mL). Results were expressed in milligrams of rutin equivalents per Gram of extract (mg RE/g extract).

### 2.6 Determination of carotenoid content

The method described by Sass-Kiss et al. (2005) is used to determine the carotenoid content. Approximately 20 mL of a hexane-acetone-ethanol mixture (2:1:1) solvent were added to 2 g of the dried and ground stamen sample. The solution is agitated in the dark at 170 rpm for 30 min. The hexane phase is recovered, and the lower phase is extracted a second time using 10 mL of hexane. Subsequently, both filtrates are combined, and absorbances are measured at 420 nm. Using the calibration curve of β-carotene, carotenoid concentrations are expressed in milligrams equivalent to β-carotene per 100 g of dry matter (mg carotene equivalents/100 g DM).

### 2.7 High-performance liquid chromatography analysis of *Crocus sativus* stamens extracts

Hydroethanolic and hydromethanolic extracts of *C. sativus* stamens were analyzed by high-performance liquid chromatography (HPLC) using the Waters AllienceTM e2695 XC system equipped with a 2,998 photodiode array detector. The sample (10 mg/mL in methanol) was injected into a reversed-phase C18 column (5 μm, 250 mm × 4.6 mm) at a flow rate of 1 mL/min The following gradient of mobile phase A (2% acetic acid in water) and mobile phase B (methanol) was used for compound separation: Initial 80% (A), 20% (B); 20 min 100% (B); 25 min 100% (B); 30 min 50% (A), 50% (B); 35 min 80% (A), 20% (B) (Ouahhoud et al., 2023). Injection volume was 10 µL and UV detection was performed in the 210–400 nm range. The phenolic compounds present in each extract were tentatively identified by comparison with their retention time and UV against standard commercial norms. The analysis of crocetin esters was performed under the same conditions and with the same mobile phase. The DAD detector was set to 440 nm for the detection of crocetin esters. Identification was achieved by comparing their retention times and UV spectra with those of commercial standards, as well as referencing the study conducted by (Vignolini et al., 2008).

### 2.8 Porcine pancreatic alpha-amylase inhibitory activity

The quantification of α-amylase inhibitory activity was conducted using an assay adapted from the method developed by Daoudi et al. (2020). The reaction mixture comprised 0.2 mL of α-amylase enzyme solution (13 IU), 0.2 mL of phosphate buffer (0.02 M; pH = 6.9), and 0.2 mL of *C. sativus* stamens, their fractions, or acarbose at various concentrations. This solution was pre-incubated at 37°C for 10 min. Subsequently, 0.2 mL of a 1% starch solution in the aforementioned buffer was added, and the mixture was further incubated at 37°C for 20 min. The reaction was halted by introducing 0.6 mL of DNSA color reagent, followed by a 10-min incubation in a boiling water bath and subsequent cooling in an ice-cold water bath for a few minutes. After adding 1 mL of distilled water and diluting the reaction mixture, the absorbance was measured at 540 nm. The experiment was performed in triplicates, and the percentage of enzyme inhibition by the sample was calculated using the formula:

$$\text{Inhibitory }\text{activity }\text{percentage}=\frac{\left(\text{DO}\;\text{control}\;540\;\text{nm}\;-\;\text{DO}\;\text{Test}\;540\;\text{nm}\right)}{\;\text{DO}\;\text{Control}\;540\;\text{nm}}\times 100$$

Using the following mathematical function, the concentration of the samples that inhibit 50% (IC_50_) of α-amylase enzymatic activity is graphically determined:

$$\text{Inhibition }\text{percentage}=f\;\left(\log\text{sampleconcentration}\right).$$

### 2.9 Porcine pancreatic alpha-amylase inhibitory activity *in vivo*

#### 2.9.1 Experimental animals

Normoglycemic *Wistar* rats (♂/♀), (weight 200–250 g) from the animal house of the Biology Department, Faculty of Sciences, Oujda, Morocco, were maintained individually in wire cages at constant temperature (21°C ± 2°C) and a 12-h light/dark cycle with free access to food and water for 1 week for adaptation. The study was approved by the Faculty of Sciences at Mohammed First University, Oujda (Morocco) under the trial registration numbers 10/21-LBBEH-12 and 22/03/2021.

#### 2.9.2 Induction of diabetes

Diabetes was induced following the protocol described by Prince et al. (Prince et al., 1998) with slight modifications. A single intraperitoneal injection (i.p.) of alloxan monohydrate dissolved in fresh and cold phosphate citrate buffer (pH = 4.5) at a dose of 120 mg/kg body weight induced experimental diabetes. Rats with blood glucose levels higher than 1.25 g/L were added to the experiment 1 week later.

#### 2.9.3 Oral starch tolerance test

The rats were divided into six groups, each comprising six rats (*n* = 6).

Group 1 (control): orally received distilled water (10 mL/kg).

Group 2 (positive control): orally received acarbose (50 mg/kg, bw).

Groups 3, 4, 5, 6 (experimental groups): orally administered 250 mg/kg of plant extract, including MethOH and EthOH respectively.

Thirty minutes after the oral administration of the respective treatments, the rats were orally loaded with 2 g/kg of starch. Blood samples were collected from the tip of the tail under light anesthesia at 0, 30, 60, and 120 min after starch administration. The blood glucose concentration was determined using the glucose peroxidase method (Daoudi et al., 2020).

The calculation of the area under the curve (AUC) only included the increase in blood glucose levels throughout the course of the 120-min test (AUC0-120 min).

### 2.10 Molecular docking protocol

A molecular docking analysis was executed following the detailed guidelines outlined in reference (Kandsi et al., 2022). The crystalline structure of α-amylase (PDB ID: 1SMD) was sourced from the RCSB Protein Data Bank, established at the Brookhaven National Laboratory in 1971 (accessed on 2 January 2024). Using AutoDock Tools, water molecules were eliminated, and polar hydrogens, along with Kollman charges, were introduced. Subsequently, co-crystallized ligands were excluded, and the protein was formatted in “pdbqt.” Ligands, obtained in “sdf 3D” format from PubChem (accessed on 2 January 2024), underwent conversion to “pdb” files with the assistance of PyMol. Subsequently, AutoDock Tools (version 1.5.6) was employed to generate the final pdbqt files for the ligands. To accommodate the active binding site, the grid box for the docking search space was expanded. The α-amylase enzyme’s grid box dimensions were set at a uniform grid size of 40, with center coordinates (x, y, and z) at 17.39, 61.804, and 15.925. Docking simulations yielded data on the complexes, represented in terms of kcal/mol ΔG binding energy values. Acarbose served as the native ligand in this computational investigation. Analysis of protein-ligand binding interactions was conducted, and 2D molecular interaction diagrams were crafted using Discovery Studio 4.1 by Dassault Systems Biovia, headquartered in San Diego, CA, United States. This systematic approach enhances the robustness of the molecular docking study, ensuring a comprehensive exploration of α-amylase interactions with diverse ligands within its active binding site.

### 2.11 Prediction of the toxicity analysis (Pro−Tox II)

Utilizing established methodologies, the web-based tool Pro-Tox (https://toxnew.charite.de/protox_II/, accessed on 9 January 2023) played a pivotal role in evaluating potential toxicity (Banerjee et al., 2018; Ouahabi et al., 2023). This application adopts a comprehensive methodology by harnessing advanced statistical techniques. Its primary objective is to establish correlations between the chemical composition of a substance and a wide range of well-known hazardous chemicals. This proactive approach enables the anticipation of potential adverse effects or deleterious consequences on human and other biological systems. Pro-Tox significantly advances our understanding of a substance’s inherent toxicity by offering valuable insights into various factors. These encompass LD_50_ values, toxicity stratification, and a comprehensive range of toxicological outcomes, including hepatotoxicity, carcinogenicity, immunotoxicity, mutagenicity, and cytotoxicity (Banerjee et al., 2018). By delivering this pertinent information, Pro-Tox emerges as an indispensable tool in the assessment of a material’s potential harm, thereby contributing to informed decision-making in diverse fields (Banerjee et al., 2018).

### 2.12 Statistical analysis

The results obtained were presented as mean ± Standard Mean Error (SEM) and were analyzed, using the one-factor ANOVA (Analysis of Variance) test and followed by Tukey’s multiple comparison post-test with significance levels of *p* < 0.05, *p* < 0.01 and *p* < 0.001.

## 3 Results

### 3.1 Yields

The extraction procedure was used three (3) times for each plant material. The extraction yield was determined as milligrams of extracted extract per Gram of plant material, and the results were expressed as mean ± SEM. For hydroethanolic (EthOH) and hydromethanolic (MethOH) extracts of stamens, the evaluated yields were 49, 25 1, 43 mg/g and 49, 8 1, 1 mg/g, respectively (Table 1).

**TABLE 1:** Yield, total phenolic and flavonoid content of *Crocus sativus* stamens.

Extract	Extraction yield (%)	Total phenolic content (mg GAE/g)	Total flavonoid content (μg RE/g)
**MethOH**	49.8 ± 1.1	126.31 ± 0.002	243.22 ± 0.003
**EthOH**	49.25 ± 1.43	119.17 ± 0.02	655.00 ± 0.002

Values are expressed as mean SEM (n = 3). MethOH, hydromethanolic extract; EthOH, hydroethanolic extract.

### 3.2 Total phenolic and flavonoid contents

Gallic acid calibration curves were used to calculate the total phenolic and total flavonoid contents (*Y* = 0.0035X + 0.034, R2 = 0.9963) and rutin (Y = 0.0014X + 0.0082, R2 = 0.9942), respectively. The results for total phenolic and total flavonoid contents across different extracts are presented in Table 1. The findings revealed that the highest total phenolic content was observed in MethOH (126.31 ± 0.002 mg GAE/g), followed by EthOH (119.17 ± 0.02 mg GAE/g). EthOH also exhibited the highest levels of total flavonoids (655.00 ± 0.002 μg RE/g), whereas the lowest level was detected in MethOH (243.22 ± 0.003 μg RE/g).

### 3.3 Carotenoid content

The carotenoid pigment extraction results were expressed as milligrams of β-carotene per Gram of *C. sativus* stamen dry matter (mg Eq β-C/g DM). Employing a β-carotene calibration curve (*Y* = 0.0134X + 0.2596, R2 = 0.9989), the determined carotenoid content was 152.97 ± 4.4 mg Eq β-C/g DM.

### 3.4 High-performance liquid chromatography analysis of *Crocus sativus* stamens extracts

#### 3.4.1 Detection of phenolic compounds at wavelengths of 245–254 nm

The results of HPLC-DAD analysis of the main phenolic constituents of the hydromethanolic extract of *C. sativus* stamens, carried out at wavelength 245–254 nm, are presented in Table 2. Several peaks were identified using this analysis, with varying retention times. Kaempferol and rutin, with surface percentages of 16.16% and 13.73%, were the two most important molecules found. Three other peaks, with percentages of 5.98%, 5.32% and 2.90%, represent caffeic acid, coumarin and vanillic acid respectively.

**TABLE 2:** HPLC-DAD identification of the main constituents of hydromethanolic extract at wavelength 245–254 nm.

Peak no	Identified compound	Retention time (min)	Peak area (%)
1	Vanillic acid	7,778	2.90
2	Caffeic acid	8,802	5.98
3	Kaempferol	11,229	16.16
4	Rutin	11,573	13.73
5	Coumarin	12,240	5.32

#### 3.4.2 Detection of carotenoids at 400 nm wavelength

**FIGURE 1:**
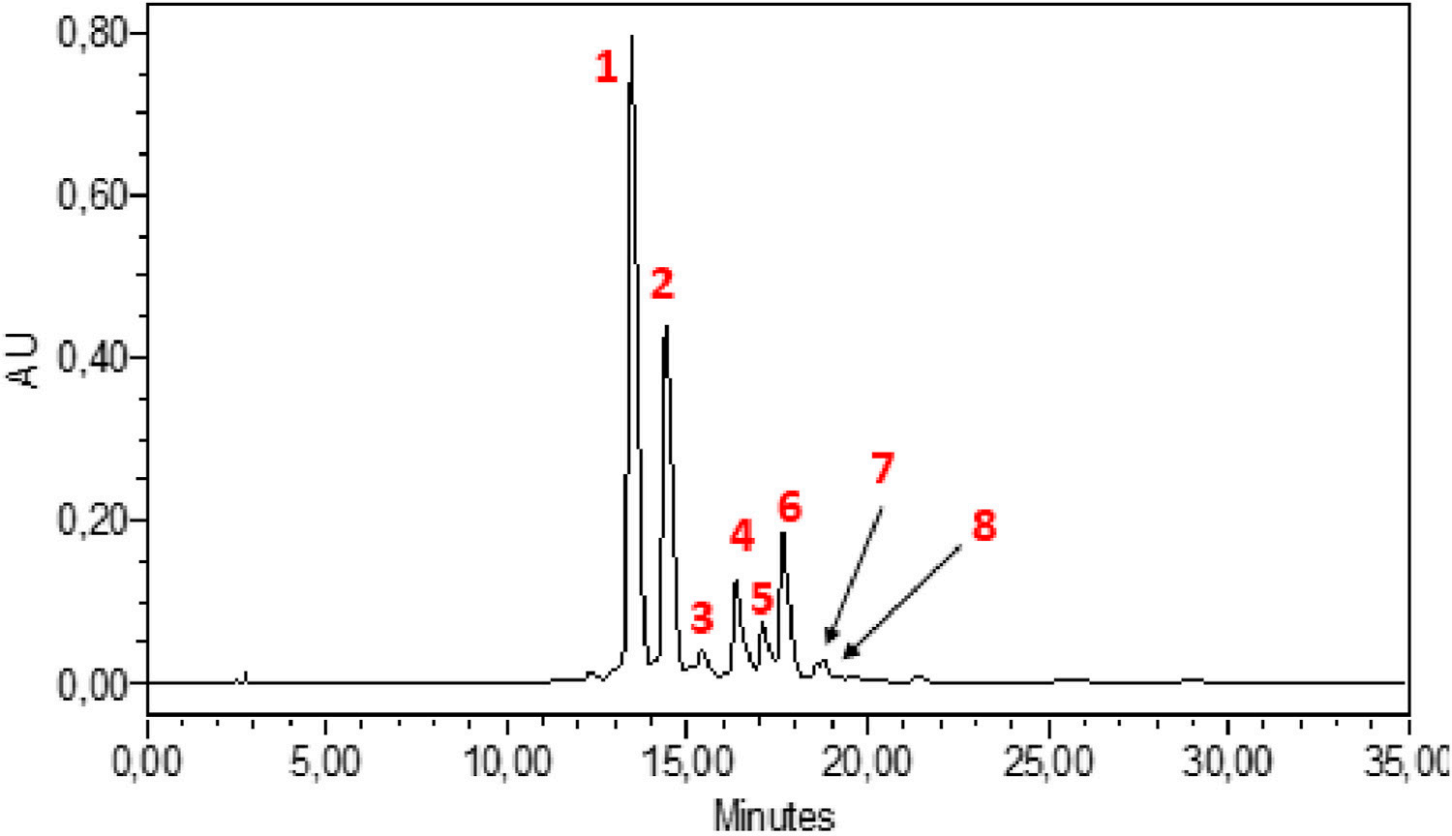
HPLC-DAD chromatogram of hydromethanolic extract at wavelength 440 nm. 1: Trans-crocin-4, 2: Trans-crocin-3, 3: Trans–crocin-2, 4: Cis-crocin-4, 5: Cis-crocin-3.6: Cis-crocin, 7: Trans-crocin-2, 8: Cis-crocin-2.

**FIGURE 2:**
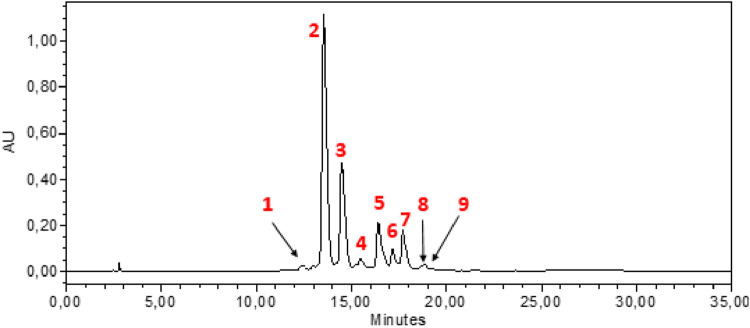
HPLC-DAD chromatogram of hydroethanolic extract at wavelength 440 nm. 1: Cis-crocin-5, 2: Trans-crocin-4, 3: Trans-crocin-3, 4: Trans–crocin-2, 5: Cis-crocin-4, 6: Cis-crocin-3, 7: Trans-crocin-1, 8: Trans-crocin, 9: Cis-crocin-2.

HPLC-DAD analysis of the hydromethanolic and hydroethanolic extracts at a detection wavelength of 400 nm enabled identification and quantification of the crocetin glycosides present in these extracts. The trans-4, trans-3, trans-2, cis-4, cis-3, cis-2 and trans-1 isomers were detected, with trans-crocin-4 and trans-crocin-3 peaking predominantly in both extracts (Figures 1, 2). The percentages of these isomers in the hydromethanolic extract are 48.22% and 26.55% respectively, and 52.92% and 21.54% respectively in the hydroethanolic extract, as shown in Tables 3, 4.

**TABLE 3:** HPLC-DAD identification of the main constituents of the hydromethanolic extract at wavelength 440 nm.

Peak no	Identified compound	Retention time (min)	Peak area (%)
1	Trans-crocin-4	13,459	48.22
2	Trans-crocin-3	14,414	26.55
3	Trans –crocin-2	15,399	1.26
4	Cis-crocin-4	16,357	7.28
5	Cis-crocin-3	17,097	3.92
6	Cis-crocin	17,648	10.73
7	Trans-crocin-2	18,581	0.54
8	Cis-crocin-2	18,789	0.96

**TABLE 4:** HPLC-DAD identification of the main constituents of the hydroethanolic extract at wavelength 440 nm.

Peak no	Identified compound	Retention time (min)	Peak area (%)
1	Cis-crocin-5	12,381	1.06
2	Trans-crocin-4	13,531	52.92
3	Trans-crocin-3	14,482	21.54
4	Trans –crocin-2	15,465	1.98
5	Cis-crocin-4	16,406	9.75
6	Cis-crocin-3	17,143	3.79
7	Trans-crocin-1	17,699	7.50
8	Trans-crocin	18,624	0.32
9	Cis-crocin-2	18,836	0.80

### 3.5 Porcine pancreatic alpha-amylase inhibitory activity

**FIGURE 3:**
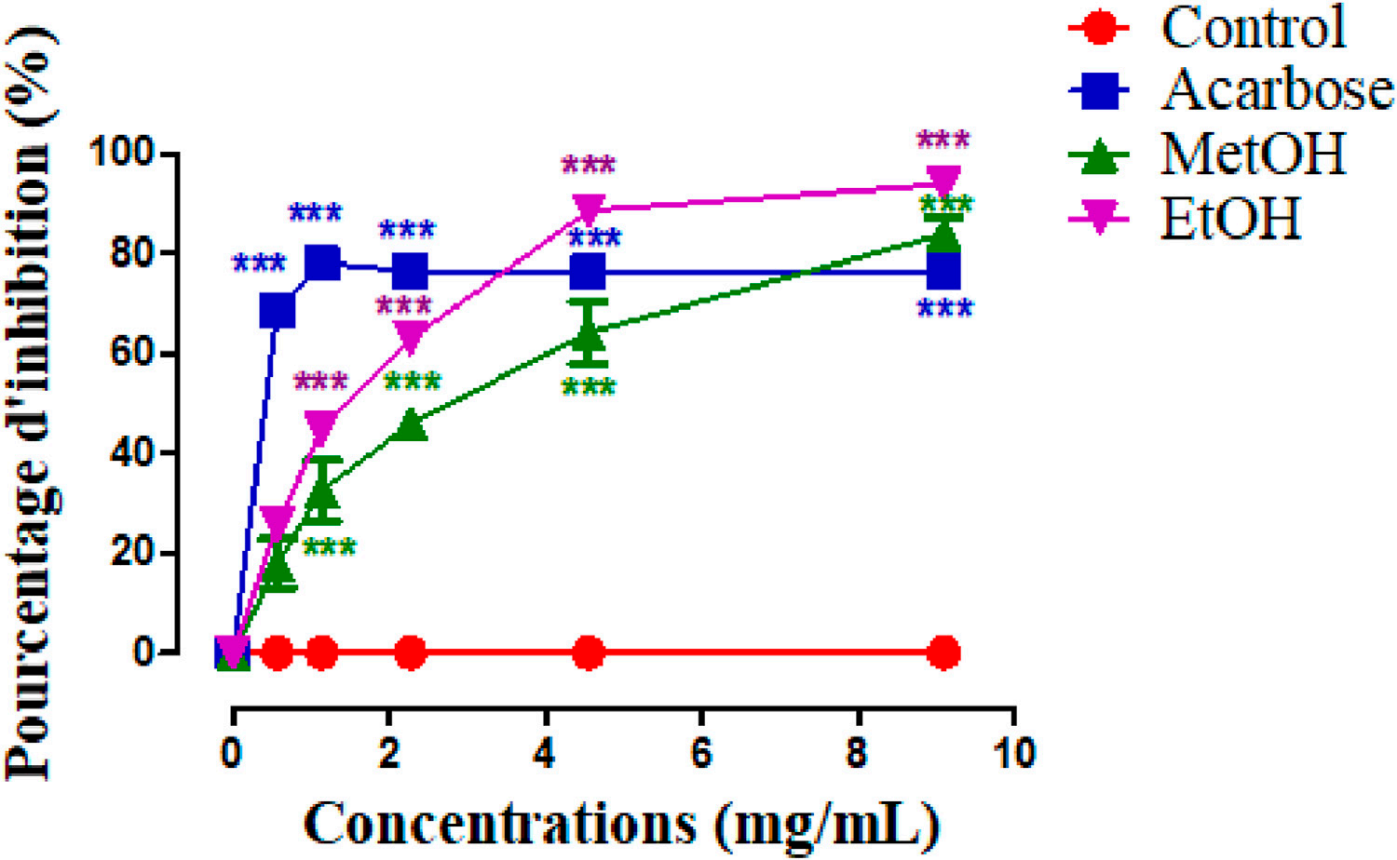
Inhibitory activity of Crocus sativus stamens extracts and acarbose (Positive control) against α-amylase at different doses. ***p < 0.001 as compared to the control. MethOH = hydromethanolic extract; EthOH = hydroethanolic extract.

Figure 3 illustrate the α-amylase inhibitory activity of stamens extract, and acarbose, which is used as a reference. The *in vitro* α-amylase inhibitory study demonstrated that both MethOH and EthOH extracts inhibit significantly the α-amylase activity. The percentage inhibition at 0.5, 1.1, 2.2, 4.5, 9.09 mg/mL concentrations of MethOH and EthOH extracts demonstrated a reduction in percentage inhibition that was dependent on concentration. Therefore, the highest tested concentration of 9.09 mg/mL exhibited a maximum inhibition of nearly 83% for Met OH and 89% for EthOH extract.

To better compare the percentages of α-amylase inhibition, we calculated the IC_50_ values for each sample (Table 5) and we found that EthOH has a higher inhibitory activity than MethOH extract (*p* < 0.001), and this effect is similar to acarbose.

**TABLE 5:** IC_50_ values of stamens extracts and acarbose in α-amylase inhibition.

Inhibitors	IC_50_ (mg/mL)
Acarbose	0.466 ± 0.03
MethOH	2.37 ± 0.20 ***
EthOH	1.37 ± 0.03 ***

Values are expressed as mean SEM (n = 3). ***p < 0.001 as compared with acarbose. MethOH, hydromethanolic extract; EthOH, hydroethanolic extract.

### 3.6 Oral strarch tolerance test of stamens extracts

**FIGURE 4:**
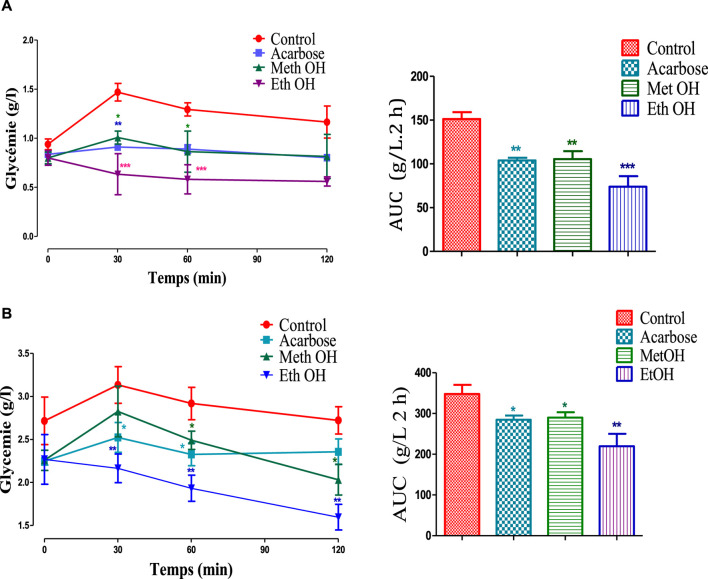
Effect of stamens extracts on glycemia following starch overload in normal **(A)** and diabetic rats **(B)**, as well as the area under the curves (AUC) (n = 6). *p < 0.05; **p < 0.01; ***p < 0.001 as compared to the control. MethOH: hydroMethanolic extract and EthOH: hydroethanolic extract.

The graph presented in Figure 4 shows the effect of stamens MethOH and EthOH extracts on blood sugar after oral starch overload in normal and alloxane-diabetic rats. The results showed that the glycemia increases from 0.94 to 1.47 g/L in the normal control group (A) and from 2.71 to 3.13 g/L in diabetic control group (B), after oral administration with starch. Whereas in the presence of MethOH and EthOH at a dose of 250 mg/kg, postprandial glycaemia is significantly reduced at time *t* = 30 min and *t* = 60 min compared to the control group. However, at 120 min blood glucose showed no significant difference compared to the control in normoglycemic group, and a significant reduction in diabetic animals. Additionally, area under the curve (AUC) assessment showed that MethOH and EthOH improved significantly oral starch tolerance in normal and diabetic rats.

### 3.7 Computational analysis using molecular docking

In this study, molecular docking served as a potent tool to elucidate the potential mode of action associated with the α-amylase activities exhibited by components extracted from *C. sativus* stamens. This state-of-the-art computational method allowed for a detailed investigation of the interactions between the chemical constituents of *C. sativus* and target enzymes, offering insights into specific binding patterns and affinities.

Through the application of molecular docking, the study significantly advanced our understanding of the molecular mechanisms governing the pharmacological effects of these components. This approach provided valuable insights into how the chemical constituents from *C. sativus* stamens interact with target enzymes, contributing to a deeper comprehension of their pharmacological effects. The utilization of molecular docking in this context represents a sophisticated and effective means of unravelling the complexities of drug actions at the molecular level.

### 3.8 *In silico* α-amylase activity of stamens extract

Based on binding affinity values, the reported data show that the examined chemical compounds may exhibit either higher or lower affinity for the target than the native ligand (acarbose) (Table 6). The foundational concept supporting this observation is that a compound’s affinity tends to increase with a decrease in binding energy.

**TABLE 6:** The binding energy, hydrogen bonds, and interacting amino acids of phytochemicals present in *Crocus sativus* stamens target protein.

N^o^.	Compounds	α-Amylase protein (PDB ID: 1SMD)	
		Affinity (kcal/mol)	H-Bonding
**-**	Acarbose ^a^	−8	THR A:6, GLN A:7, GLN A:404, ARG A:252, Ser A:289, GLY A:334, Asp A:402
**1**	Vanillic acid	−5.5	TYR A:67, LYS A:68, ALLA A:128, HIS A:185
**2**	Caffeic acid	−6.3	ARG A:252, Glu A:233, His A:299, TRP A: 59, GLN A:63
**3**	Kaempferol	−7	ASP A:317, ASP A:353
**4**	Rutin	−9.1 *	GLY A:9, ARG A:10, ARG A:252, ARG A:421, SER A:289, ASP A:402, GLN A:404, PHE A:406
**5**	Cumarin	−5.9	HIS A: 185
**6**	Crocin	−8.1*	GLY A:9, ARG A:10, ARG A:252, ARG A:421, SER A:289, ASP A:402, GLN A:404, PHE A:406
**7**	Crocin-1	−7.2	ARG A:319, LYS A: 322, TRP A:388, ARG A:389, GLN A:390, ASN A:481
**8**	Crocin-2	−6.5	ILE A:148, TYR A:151, THR A:163, GLN A:302, ILE A:312, ASP A:317, ARG A:346
**9**	Crocin-3	−7.1	ASP A:356, QV A:4,500
**10**	Crocin-4	−7.1	LYS A:200, ASN A:301, ASP A:317

^a^ acarbose, a native ligand of α-amylase; * The potent ligands in comparison of the native ligand.

The bold values indicate that two molecules, rutin and crocin, exhibit significantly lower free binding energy values compared to the natural ligand.

**FIGURE 5:**
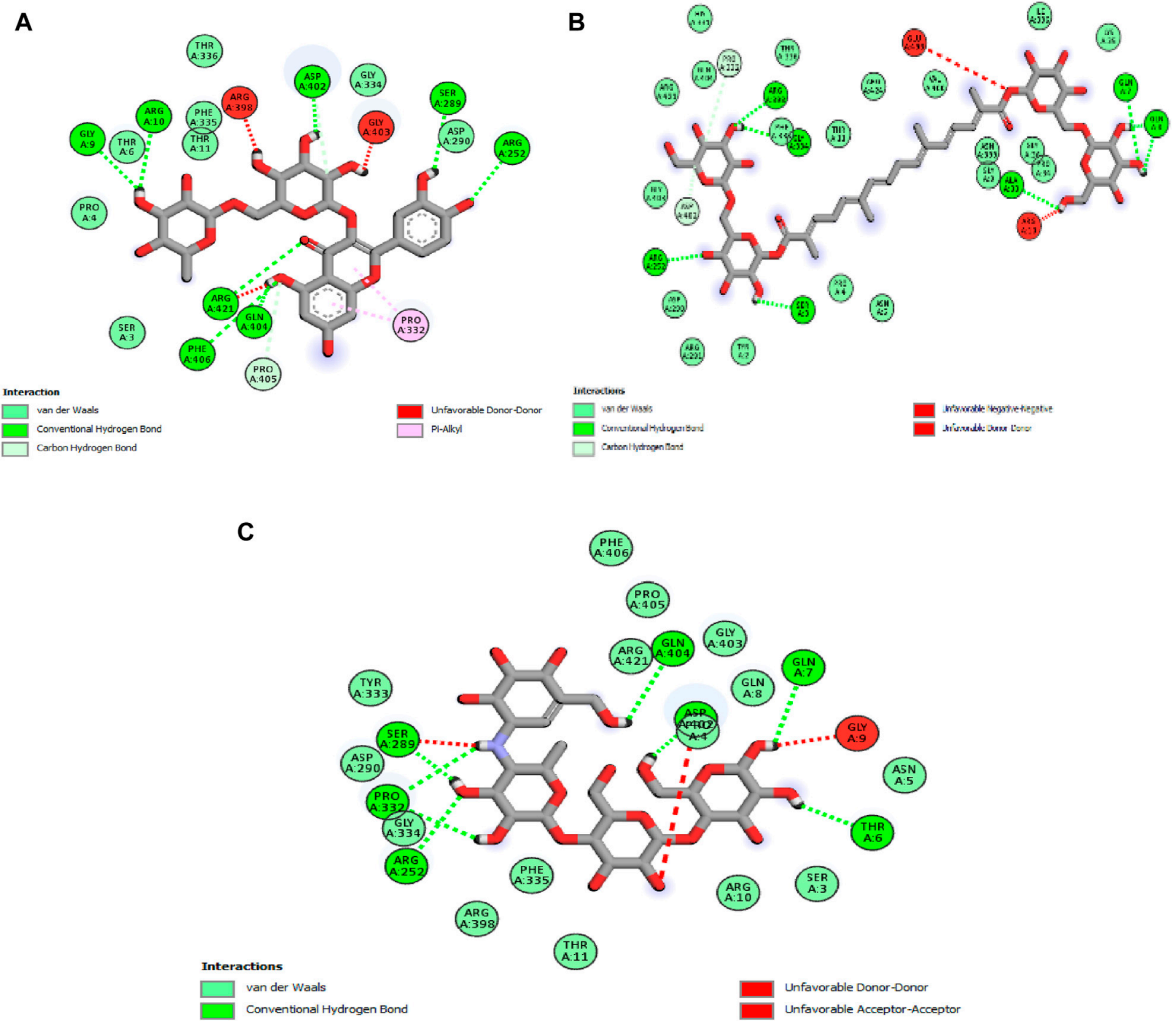
2D diagrams illustrating the interactions with amino acid residues for two potent compounds identified in stamens, **(A)** Rutin, **(B)** Crocin, and the native ligand, acarbose **(C)**.

The analysis reveals that, in this case, two molecules, rutin and crocin, exhibit lower free binding energy values than the natural ligand. Specifically, Table 6 presents the values of rutin and crocin as −9.1 and −8.1 kcal/mol, respectively. Notably, amino acid residues GLY A:9, ARG A:10, ARG A:252, ARG A:421, SER A:289, ASP A:402, GLN A:404, and PHE A:406 are involved in forming eight common hydrogen bonds with rutin. Conversely, crocin engages amino acid residues to form seven typical hydrogen bonds, as listed in Figure 5: GLY A:9, ARG A:10, ARG A:252, ARG A:421, SER A:289, ASP A:402, GLN A:404, and PHE A:406.

The outcomes of this *in silico* investigation strongly suggest that the interactions between these molecules and the designated amino acid residues are likely responsible for the observed antihyperglycemic effects and the inhibition of pancreatic α-amylase. This provides a molecular basis for understanding the pharmacological effects associated with these compounds.

### 3.9 *In silico* toxicity prediction (using Pro-Tox II)

Table 7 presents a comprehensive toxicological evaluation of prominent compounds identified in stamens extracts (MehtOH and EthOH), including vanillic acid, caffeic acid, kaempferol, rutin, coumarin, crocine, crocine 1, crocine 2, crocine 3, and crocine 4. The table includes anticipated LD_50_ values (expressed in mg/kg), categorization according to GHS hazard classes, and respective probabilities for various toxic endpoints. Insights drawn from LD_50_ values highlight those compounds 2, 3, 6, 7, 8, 9, and 10 exhibit low acute toxicity, in contrast to compound 5 (coumarin), which demonstrates high acute toxicity. Hazard classifications within GHS ranging from III to VI predominantly place the compounds in hazard classes V and VI, indicating low to moderate toxicity. While most compounds in the extracts show low toxicity, some constituents may pose risks to human health. Anticipated toxicological endpoints shed light on potential health implications, encompassing hepatotoxicity, mutagenicity, carcinogenicity, immunotoxicity, and cytotoxicity. Notably, compounds three and four exhibit an increased probability of immunotoxicity. Compound 2 stands out as a potential candidate for increased carcinogenicity, while compound 5 presents moderate probabilities of carcinogenic and cytotoxic potential.

**TABLE 7:** Prediction of toxicity, and the toxic endpoints of the major compounds found in MethOH and EthOH extracts of stamens. (1) Vanilc acid, (2) Caffeic acid, (3) Kaempferol, (4) Rutin, (5) Cumarin, (6) Crocin, (7) Crocin 1, (8) Crocin 2, (9) Crocin 3, (10) Crocin 4.

N	Predicted LD5	Class	Hepatotoxicity		Cytotoxicity		Immunotoxicity		Mutagenicity		Carcinogenicity	
	(mg/kg)		Predi	Prob	Predi	Predi	Predi	Prob	Predi	Prob	Predi	Prob
**1**	2000	IV	Ina	0.55	Ina	0.91	Ina	0.99	Ina	0.96	Ina	0.64
**2**	2,980	V	Ina	0.57	Ina	0.86	Ina	0.50	Ina	0.98	Act	0.78
**3**	3,919	V	Ina	0.96	Ina	0.88	Act	0.91	Ina	0.51	Ina	0.61
**4**	5,000	V	Ina	0.80	Ina	0.64	Act	0.96	Ina	0.88	Ina	0.91
**5**	196	III	Ina	0.96	Act	0.55	Ina	0.97	Ina	0.53	Act	0.83
**6**	8,000	VI	Ina	0.72	Ina	0.79	Ina	0.97	Ina	0.96	Ina	0.91
**7**	8,000	VI	Ina	0.72	Ina	0.79	Ina	0.99	Ina	0.96	Ina	0.91
**8**	8,000	VI	Ina	0.72	Ina	0.79	Act	0.99	Ina	0.96	Ina	0.91
**9**	8,000	VI	Ina	0.72	Ina	0.79	Ina	0.95	Ina	0.96	Ina	0.91
**10**	8,000	VI	Ina	0.77	Ina	0.81	Ina	0.96	Ina	0.82	Ina	0.84

In essence, the study on *C. sativus* stamens extracts, specifically MethOH and EthOH extract, provides crucial insights into the health implications of key compounds. These findings contribute significantly to the safe utilization of these agents. However, ongoing research is essential to fully grasp the toxicity profiles and potential risks associated with human health. The toxicological assessment highlights varying levels of toxicity among major compounds, emphasizing the importance of continued exploration to optimize benefits while minimizing health risks.

## 4 Discussion

The quantitative study of *C. sativus* stamens extracts was carried out using spectrophotometric assays, with the aim of determining the content of total polyphenols, flavonoids and carotenoids. The results obtained showed that the two extracts studied contained varying quantities of these bioactive molecules. The hydroethanol extract contained almost twice as many flavonoids as the hydromethanol extract. This is attributed to the fact that ethanol is a more suitable solvent for flavonoid extraction (Gómez-Caravaca et al., 2006). HPLC-DAD chromatograms of the stamen extracts revealed the presence of various flavonoids with distinct retention times. Kaempferol and rutin were the most abundant flavonoids in the hydromethanolic extracts.

The primary bioactive components of the phenolic group identified in ethanolic extracts of dried stamens were gallic acid (3.10 mg), syringic acid (0.20 mg), and vanillic acid (0.13 mg). In the ethanolic extracts of dried stamens, the key bioactive components of the flavonoid group included quercetin (2.13 mg g/L), pyrogallol (1.73 mg), kaempferol (1.20 mg), rutin (0.20 mg), and tricin (0.10 mg) (Mahood et al., 2023). The findings of Mottaghipisheh et al. align with our own results for stamen samples. Three flavonol glycosides, specifically quercetin-3-O-sophoroside, kaempferol-3-O-glucoside, and kaempferol-3-O-sophoroside, were identified as the main components. The amount of kaempferol-3-O-glucoside, the most abundant compound, ranged from 1.72 to 7.44 mg/g (Mottaghipisheh et al., 2020).

The total carotenoid content was determined in *C. sativus* stamens for the first time using the spectrophotometric method, and the results showed that the stamens are rich in carotenoids with a quantity of 152.97 ± 4.40 mg/g DM. Of all known species of the Crocus genus, *C. sativus* is particularly valued for its high levels of carotenoids (Ahrazem et al., 2010). Crocins, and crocetin are the main carotenoids characteristic of saffron stigmas (Javadi, Sahebkar, and Emami, 2013; Mottaghipisheh et al., 2020). Crocetin glycosides are responsible for the yellow color of stamens, their UV-Vis spectra characterized by an absorption maximum at around 440–460 nm depending on the molecule. UV-visible spectra can be used to differentiate between cis and trans crocetin, since the cis isomer absorbs at 326 and 440 nm, while the trans isomer absorbs only at 440 nm (Tarantilis, Tsoupras, and Polissiou, 1995). In general, trans isomers have a higher polarity than cis isomers. Nine crocetin glycosides were identified from the results of HPLC-DAD performed, of which trans-crocetin-4, trans-crocetin-3, cis-crocetin-4 ester, trans-crocetin-2 and cis-crocetin-3 ester were the most abundant crocetin derivatives, followed by cis-crocetin, trans-crocetin and trans-crocetin. This is consistent with the results of Vignolini et al. (Vignolini et al., 2008) who also identified 12 crocetin glycosides in *C. sativus* stamens. Crocins and crocetin, which are linked to saffron’s coloring properties, are derived from zeaxanthin. Anuar et al. (Anuar et al., 2018) identified crocins, crocetin, and zeaxanthin in both stigmas and stamens. Crocin was found at a higher detectable level in stigmas compared to crocetin and zeaxanthin. Additionally, stamens exhibited a higher concentration of crocetin compared to stigmas, with values of 17.19 mg/g and 7.52 mg/g, respectively.

Type 2 diabetes exhibits a heterogeneous nature, characterized by the interplay of defects in both insulin secretion and action. This deficiency leads to elevated blood glucose levels, causing damage to various bodily systems (Lin and Sun, 2010). Managing postprandial blood glucose is crucial for diabetes control, and inhibiting digestive enzymes, particularly α-glucosidase and α-amylase, is considered a promising strategy for reducing postprandial blood glucose levels and a significant target in diabetes management (Bischoff, 1994; Lebovitz, 1995). While synthetic glucosidase inhibitors like acarbose are available, there is a growing interest in exploring natural sources as nutritional alternatives due to their potential for minimal side effects and lower therapy costs (Van de Laar et al., 2005). Acarbose, a pseudo-tetrasaccharide of microbial origin, effectively inhibits various brush-border enzymes and pancreatic α-amylase (Bischoff, 1994). Despite its efficacy, acarbose is associated with side effects like diarrhea and flatulence, necessitating the exploration of alternative food-based options (Wang et al., 1999). With the renewed focus on nutritional therapy and functional foods, there is an increased interest in exploring preventive and modulatory effects on diabetes and obesity (Evert et al., 2014).

A few papers have described antidiabetic effect of *C. sativus* by-products (Rocher et al., 1992). Nevertheless, none of the previous studies determined the glucosidase and amylase inhibitory activities of stamens. Furthermore, the reported work has focused on stigmas and tepals. In this context, the objective of our study was to investigate the impact of *C. sativus* stamen extracts on postprandial hyperglycemia. Our study based on the *in vitro* evaluation of the effect of extracts on the activity of the enzyme α-amylase. However, *in vitro* analyses are not sufficient and must be confirmed by other tests. For that, we have confirmed these activities *in vivo* in normal and in alloxan-diabetic *Wistar* rats. The results of this experiment prove that the EthOH and MethOH extracts exhibited an *in vitro* inhibiting potential on the pancreatic enzyme α-amylase especially EthOH has a higher inhibitory activity than MethOH extract. The anti-enzymatic activity of stamens is probably related in part to its phenolic content. Stamen extracts were shown to have promising levels of phenolic compounds. The metabolites of the extracts were able to bind to either the free enzyme or the enzyme-substrate complex.

The *in vivo* investigation demonstrates that shortly after starch administration, both MethOH and EthOH extracts were effective in reducing blood sugar levels in both normal and diabetic rats, validating the *in vitro* findings. Furthermore, the assessment of the area under the curve (AUC) revealed a significant improvement in oral starch tolerance in both normal and diabetic rats treated with MethOH and EthOH extracts of *C. sativus* stamens.

The application of molecular docking in this study has unveiled the potential mode of action of α-amylase activities exhibited by components from stamens. By exploring the interactions between these components and target enzymes, the study deepens our understanding of the molecular pathways governing the pharmacological effects of these compounds. The *in silico* analysis, based on binding affinity values, highlights rutin and crocin as promising molecules with lower free binding energy than the natural ligand. Notably, hydrogen bond formations between these molecules and specific amino acid residues around the active sites of α-amylase provide insights into their antihyperglycemic effects and pancreatic α-amylase inhibition. The findings obtained through *in silico* analysis align with the research conducted by Birari et al. (Birari and Bhutani, 2007), demonstrating the inhibitory capabilities of crocin on the alpha-amylase enzyme. Bhat et al. (Singla and Bhat, 2011) reported that crocin can effectively inhibit both α-amylase and α-glucosidase activities, resulting in a reduction in blood sugar levels. Similarly, rutin, a flavonoid glycoside, demonstrated significant *in vitro* inhibitory activity against the α-amylase enzyme as demonstrated by Dubey et al. (Dubey et al., 2017).

## 5 Conclusion

The results of the current study indicate that various extracts and fractions derived from *C. sativus* stamens exhibit a noteworthy inhibitory effect on the α-amylase digestive enzyme. The observed variability in this effect can be directly linked to the nature of the fractions and their phenolic content. Further research is warranted to validate the antidiabetic activity of these diverse fractions, paving the way for their potential use as alternatives to commercially available drugs.

## Data Availability

The raw data supporting the conclusion of this article will be made available by the authors, without undue reservation.

## References

[B1] Ahrazem O. Trapero A. Dolores Gómez M. Rubio-Moraga A. Gómez-Gómez L. (2010). Genomic analysis and gene structure of the plant carotenoid dioxygenase 4 family: a deeper study in Crocus sativus and its allies. Genomics 96 (4), 239–250. 10.1016/j.ygeno.2010.07.003 20633636

[B2] Anuar N. Taha R. M. Mahmad N. Othman R. (2018). Identification of crocin, crocetin and zeaxanthin in Crocus sativus grown under controlled environment in Malaysia. Pigment Resin Technol. 47 (6), 502–506. 10.1108/prt-11-2016-0107

[B3] Banerjee P. Eckert A. O. Schrey A. K. Preissner R. (2018). ProTox-II: a webserver for the prediction of toxicity of chemicals. Nucleic Acids Res. 46 (W1), W257–W263. 10.1093/nar/gky318 29718510 PMC6031011

[B4] Benjamin E. J. Salim S. V. Callaway C. W. Chamberlain A. M. Chang A. R. Cheng S. (2018). Heart disease and stroke statistics—2018 update: a report from the American heart association. Circulation 137 (12), e67–e492. 10.1161/cir.0000000000000558 29386200

[B5] Birari R. B. Bhutani K. K. (2007). Pancreatic lipase inhibitors from natural sources: unexplored potential. Drug Discov. Today 12 (19–20), 879–889. 10.1016/j.drudis.2007.07.024 17933690

[B6] Bischoff H. (1994). Pharmacology of α‐glucosidase inhibition. Eur. J. Clin. Investigation 24 (S3), 3–10. 10.1111/j.1365-2362.1994.tb02249.x 8001624

[B7] Ceriello A. Hanefeld M. Leiter L. Monnier L. Moses A. Owens D. (2004). Postprandial glucose regulation and diabetic complications. Archives Intern. Med. 164 (19), 2090–2095. 10.1001/archinte.164.19.2090 15505121

[B8] Daoudi N. E. Bouhrim M. Ouassou H. Legssyer A. Mekhfi H. Ziyyat A. (2020). Inhibitory effect of roasted/unroasted argania spinosa seeds oil on α-glucosidase, α-amylase and intestinal glucose absorption activities. South Afr. J. Bot. 135, 413–420. 10.1016/j.sajb.2020.09.020

[B9] Dubey S. Ganeshpurkar A. Ganeshpurkar A. Bansal D. Dubey N. (2017). Glycolytic enzyme inhibitory and antiglycation potential of rutin. Future J. Pharm. Sci. 3 (2), 158–162. 10.1016/j.fjps.2017.05.005

[B10] Evert A. B. Boucher J. L. Cypress M. Dunbar S. A. Franz M. J. Mayer-Davis E. J. (2014). Nutrition therapy recommendations for the management of adults with diabetes. Diabetes Care 37 (Suppl_1), S120–S143. 10.2337/dc14-s120 24357208

[B11] Fan W. Glovaci D. Wong N. (2019). Epidemiology of diabetes mellitus and cardiovascular disease. Heart Dis. Prev. Program 21 (4), 21. University of California. 10.1007/s11886-019-1107-y 30828746

[B12] Gómez-Caravaca A. M. Gómez-Romero M. Arráez-Román D. Segura-Carretero A. Fernández-Gutiérrez A. (2006). Advances in the analysis of phenolic compounds in products derived from bees. J. Pharm. Biomed. Analysis 41 (4), 1220–1234. 10.1016/j.jpba.2006.03.002 16621403

[B13] Javadi B. Sahebkar A. Emami S. A. (2013). A survey on saffron in major islamic traditional medicine books. Iran. J. Basic Med. Sci. 16 (1), 1–11. 23638288 PMC3637900

[B14] Kandsi F. Elbouzidi A. Lafdil F. Z. Meskali N. Ali A. Mohamed A. (2022). Antibacterial and antioxidant activity of dysphania ambrosioides (L.) mosyakin and clemants essential oils: experimental and computational approaches. Antibiotics 11 (4), 482. 10.3390/antibiotics11040482 35453233 PMC9031865

[B15] Khan M. Hanif M. A. Adnan Ayub M. Jilani M. I. Chatha S. A. S. (2020). “Chapter 43 - saffron,” in Medicinal plants of south asia byrne. Editors Asif Hanif M. Nawaz H. Mumtaz Khan M. Hugh J. B. T. (Elsevier), 587–600. 10.1016/B978-0-08-102659-5.00043-4

[B16] Khoulati A. Ouahhoud S. Mamri S. Alaoui K. Lahmass I. Choukri M. (2019). Saffron extract stimulates growth, improves the antioxidant components of solanum lycopersicum L., and has an antifungal effect. Ann. Agric. Sci. 64 (2), 138–150. 10.1016/j.aoas.2019.10.002

[B17] Lamien-Meda A. Lamien C. E. Compaoré M. M. Y. Meda R. N. T. Kiendrebeogo M. Zeba B. (2008). Polyphenol content and antioxidant activity of fourteen wild edible fruits from Burkina Faso. Molecules 13 (3), 581–594. 10.3390/molecules13030581 18463567 PMC6245336

[B18] Lebovitz H. E. (1995). A new oral therapy for diabetes management: alpha-glucosidase inhibition with acarbose. Clin. Diabetes 13 (6), 99–104.

[B19] Lin Yi Sun Z. (2010). Current views on type 2 diabetes. J. Endocrinol. 204 (1), 1–11. 10.1677/joe-09-0260 19770178 PMC2814170

[B20] Lotfi Z. Aboussaleh Y. Sbaibi R. Achouri I. Benguedour R. (2017). The overweight, the obesity and the glycemic control among diabetics of the provincial reference center of diabetes (CRD), Kenitra, Morocco. Pan Afr. Med. J. 27, 189. 10.11604/pamj.2017.27.189.9535 28904714 PMC5579421

[B21] Mahood H. E. Ali A. D. Sarropoulou V. Tzatzani T.-T. (2023). Extraction of phenolic and flavonoid compounds and evaluation of their antioxidant activity in saffron anthers (Crocus sativus L.). Not. Sci. Biol. 15 (4), 11640. 10.55779/nsb15411640

[B22] Mamri S. Elhouda Daoudi N. Marghich M. Ouahhoud S. Khoulati A. Choukri M. (2022). Protective effect of Crocus sativus stamens extract on gentamicin-induced nephrotoxicity and oxidative damage in rat kidney. J. Exp. Biol. Agric. Sci. 10 (1), 73–82. 10.18006/2022.10(1).73.82

[B23] Montoro P. Maldini M. Luciani L. Tuberoso C. I. G. Congiu F. Pizza C. (2012). Radical scavenging activity and LC-MS metabolic profiling of petals, stamens, and flowers of Crocus sativus L. J. Food Sci. 77 (8), C893–C900. 10.1111/j.1750-3841.2012.02803.x 22809329

[B24] Mottaghipisheh J. Sourestani M. M. Kiss T. Horváth A. Tóth B. Ayanmanesh M. (2020). Comprehensive chemotaxonomic analysis of saffron Crocus tepal and stamen samples, as raw materials with potential antidepressant activity. J. Pharm. Biomed. Analysis 184, 113183. 10.1016/j.jpba.2020.113183 32105944

[B25] Nair S. S. Kavrekar V. Mishra A. (2013). *In vitro* studies on alpha amylase and alpha glucosidase inhibitory activities of selected plant extracts. Eur. J. Exp. Biol. 3 (1), 128–132.

[B26] N’doua L. A. R. Abo K. J. C. Aoussi S. Kouakou L. K. Ehile E. E. (2016). Aqueous extract of rauwolfia vomitoria afzel (apocynaceae) roots effect on blood glucose level of normoglycemic and hyperglycemic rats. Am. Acad. Sci. Res. J. Eng. Technol. Sci. 20 (1), 66–77.

[B27] Ouahabi S. Loukili El H. Elbouzidi A. Taibi M. Bouslamti M. Nafidi H.-A. (2023). Pharmacological properties of chemically characterized extracts from mastic tree: *in vitro* and *in silico* assays. Life 13 (6), 1393. 10.3390/life13061393 37374175 PMC10301720

[B28] Ouahhoud S. Khoulati A. Kadda S. Bencheikh N. Mamri S. Ziani A. (2022). Antioxidant activity, metal chelating ability and DNA protective effect of the hydroethanolic extracts of Crocus sativus stigmas, tepals and leaves. Antioxidants 11 (5), 932. 10.3390/antiox11050932 35624796 PMC9137568

[B29] Ouahhoud S. Mohamed M. Makrane H. Ahmed K. Khoulati A. Mamri S. (2023). *In vitro* assessment of myorelaxant and antispasmodic effects of stigmas, tepals, and leaves hydroethanolic extracts of Crocus sativus. J. Food Biochem. 2023, 1–12. 10.1155/2023/4165305

[B30] Ozougwu J. C. Obimba K. C. Belonwu C. D. Unakalamba C. B. (2013). The pathogenesis and pathophysiology of type 1 and type 2 diabetes mellitus. J. Physiol. Pathophysiol. 4 (4), 46–57. 10.5897/jpap2013.0001

[B31] Prince P. S. M. Menon V. P. Pari L. (1998). Hypoglycaemic activity of syzigium cumini seeds: effect on lipid peroxidation in alloxan diabetic rats. J. Ethnopharmacol. 61 (1), 1–7. 10.1016/s0378-8741(98)00002-6 9687076

[B32] Rocher A. Colilla F. Ortiz M. L. Mendez E. (1992). Identification of the three major coeliac immunoreactive proteins and one α‐amylase inhibitor from oat endosperm. FEBS Lett. 310 (1), 37–40. 10.1016/0014-5793(92)81141-8 1526282

[B33] Sabir O. Iliass L. Mohamed B. Amine K. Assia S. Redouane B. (2018). Antidiabetic effect of hydroethanolic extract of Crocus sativus stigmas, tepals and leaves in streptozotocin-induced diabetic rats. Physiology Pharmacol., 0. 10.32598/ppj.25.2.30

[B34] Sass-Kiss A. Kiss J. Milotay P. Kerek M. M. Toth-Markus M. (2005). Differences in anthocyanin and carotenoid content of fruits and vegetables. Food Res. Int. 38 (8–9), 1023–1029. 10.1016/j.foodres.2005.03.014

[B35] Schmidt M. Betti G. Hensel A. (2007). Saffron in phytotherapy: pharmacology and clinical uses. Wien. Med. Wochenschr. 157 (13), 315–319. 10.1007/s10354-007-0428-4 17704979

[B36] Singla R. K. Bhat G. V. (2011). Crocin: an overview. Indo Glob. J. Pharm. Sci. 1 (4), 281–286. 10.35652/igjps.2011.27

[B37] Smolskaite L. Talou T. Fabre N. Venskutonis P. R. (2011). “Valorization of saffron industry by-products: bioactive compounds from leaves,” in Innovations for Food Science and Production, Proceedings of the 6th Baltic Conference on Food Science and Technology FOODBALT-2011, Jelgava, Latvia, May 5–6, 2011, 5–6.

[B38] Tarantilis P. A. George T. Polissiou M. (1995). Determination of saffron (Crocus sativus L.) components in crude plant extract using high-performance liquid chromatography-UV-visible photodiode-array detection-mass spectrometry. J. Chromatogr. A 699 (1–2), 107–118. 10.1016/0021-9673(95)00044-n 7757208

[B39] Tundis R. Loizzo M. R. Menichini F. (2010). Natural products as alpha-amylase and alpha-glucosidase inhibitors and their hypoglycaemic potential in the treatment of diabetes: an update. Mini Rev. Med. Chem. 10 (4), 315–331. 10.2174/138955710791331007 20470247

[B40] Van de Laar F. A. Lucassen P. L. Akkermans R. P. Van de Lisdonk E. H. Rutten G. E. Van Weel C. (2005). Alpha-glucosidase inhibitors for type 2 diabetes mellitus (cochrane review). Cochrane Database Syst. Rev. 18 (2). 10.1002/14651858.CD003639.pub2 PMC8406605 15846673

[B41] Vignolini P. Heimler D. Pinelli P. Ieri F. Sciullo A. Romani A. (2008). Characterization of by-products of saffron (Crocus sativus L.) production. Nat. Product. Commun. 3 (12), 1934578X0800301. 10.1177/1934578x0800301203

[B42] Wang P.-Yu Kaneko T. Wang Y. Sato A. (1999). Acarbose alone or in combination with ethanol potentiates the hepatotoxicity of carbon tetrachloride and acetaminophen in rats. Hepatology 29 (1), 161–165. 10.1002/hep.510290109 9862862

[B43] Zhishen J. Tang M. Wu J. (1999). The determination of flavonoid contents in mulberry and their scavenging effects on superoxide radicals. Food Chem. 64 (4), 555–559. 10.1016/s0308-8146(98)00102-2

