# Supplementary material for: Comprehensive analysis of bioactive compounds in Crocus sativus stamens extracts by HPLC-DAD: investigating antidiabetic activity via in vitro, in vivo, and molecular docking simulation
Source: Front Chem. 2024 Jul 12;12:1419120. doi: 10.3389/fchem.2024.1419120 (PMC11272647; doi:10.3389/fchem.2024.1419120)
Supplement: Supplementary file 1 [file Image1.pdf]

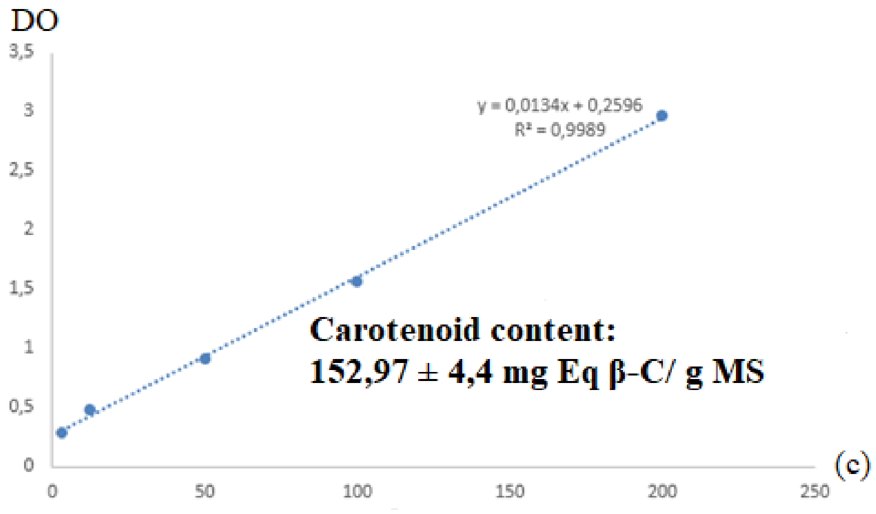

Figure. Calibration range curve for β-carotene and carotenoid levels in *Crocus sativus* stamens.
